# Association of aescin with β- and γ-cyclodextrins studied by DFT calculations and spectroscopic methods

**DOI:** 10.3762/bjnano.8.37

**Published:** 2017-02-03

**Authors:** Ana I Ramos, Pedro D Vaz, Susana S Braga, Artur M S Silva

**Affiliations:** 1 CICECO, Complexo de Laboratórios Tecnológicos, Campus Universitário de Santiago, 3810-193 Aveiro, Portugal; 2 Current affiliation: INEGI-FEUP Faculty of Engineering of the University of Porto, Rua Dr. Roberto Frias, 4200-465, Porto, Portugal; 3 CQB, Departamento de Química e Bioquímica, Faculdade de Ciências da Universidade de Lisboa, 1749-016 Lisboa, Portugal; 4 ISIS Neutron & Muon Source, Rutherford Appleton Laboratory, Chilton, Didcot, Oxfordshire OX11 0QX, United Kingdom; 5 QOPNA, Departamento de Química, Universidade de Aveiro, Campus de Santiago, 3810-193 Aveiro, Portugal

**Keywords:** aescin, cyclodextrin inclusion, DFT, ^1^H NMR, ROESY

## Abstract

**Background:** Aescin, a natural mixture of saponins occurring in *Aesculus hippocastanum*, exhibits important flebotonic properties, being used in the treatment of chronic venous insufficiency in legs. The inclusion of aescin into cyclodextrins (CDs) is a technical solution for its incorporation into the textile of stockings, but details of the physicochemistry of these host–guest systems are lacking. This work investigates the inclusion of aescin into the cavities of two native cyclodextrins, β-CD and γ-CD.

**Results:** The continuous variation method applied to aqueous-phase ^1^H nuclear magnetic resonance (^1^H NMR) has demonstrated that the preferred CD/aescin inclusion stoichiometries are 2:1 with β-CD and 1:1 with γ-CD. The affinity constant calculated for γ-CD·aescin was 894 M^−1^, while for 2β-CD·aescin it was estimated to be 715 M^−1^. Density functional theory (DFT) calculations on the interaction of aescin Ib with CDs show that an inclusion can indeed occur and it is further demonstrated that the wider cavity of γ-CD is more adequate to accommodate this large guest. ROESY spectroscopy is consistent with the formation of a complex in which the triterpenic moiety of aescin is included into the cavity of γ-CD. The higher stability of this geometry was confirmed by DFT. Furthermore, DFT calculations were applied to determine the chemical shifts of the protons H3 and H5 of the CDs in the optimised structures of the inclusion complexes. The calculated values are very similar to the experimental data, validating the approach made in this study by NMR.

**Conclusion:** The combination of experimental data from aqueous-state NMR measurements and theoretical calculations has demonstrated that γ-CD is the most suitable host for aescin, although the inclusion also occurs with β-CD. The geometry of the γ-CD·aescin complex is characterised by the inclusion of the triterpene segment of aescin into the host cavity.

## Introduction

**Figure 1:**
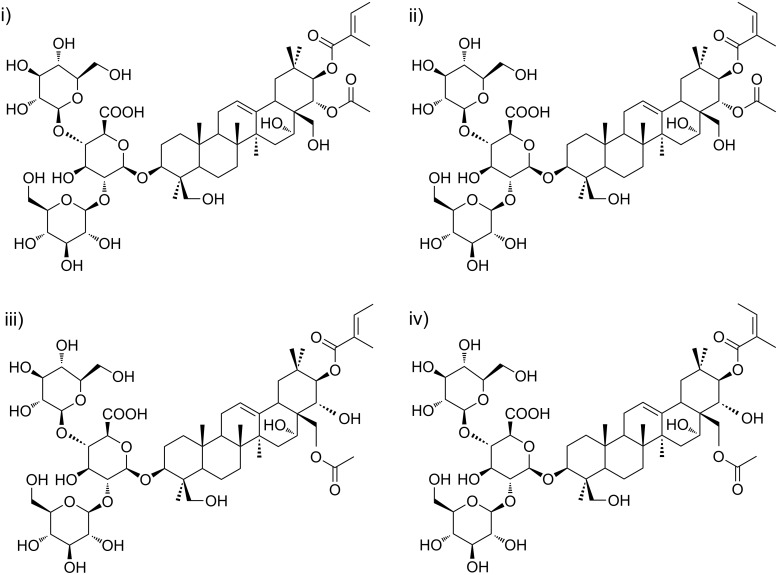
Isomeric structures for (top line) aescin Ia (i) and aescin Ib (ii) and for (bottom line) isoaescin Ia (iii) and isoaescin Ib (iv).

Aescin is the main component of the crystalline saponins obtained from the seeds of the horse chestnut tree, *Aesculus hippocastanum* (Hippocastanaceae). It is a natural mixture of acylated triterpene glycosides. In early studies, the saponins present in aescin were divided into two forms, α-aescin and β-aescin, with distinct melting point, hemolytic index, specific rotation and aqueous solubility [1]. β-Aescin was identified as the main active component and it is presently known to be not a pure compound, but rather a mixture of aescin Ia and aescin Ib (Figure 1). In turn, α-aescin was demonstrated to comprise the isomers isoaescin Ia and isoaescin Ib.

Aescin has a broad range of biological activities, the best-known being anti-oedematous, anti-inflammatory and venotonic actions [1]. In China, injectable forms of aescin are widely used in the clinic to prevent inflammatory oedema after trauma from fracture or surgery, in spite of the known side effects (phlebitis and allergic reactions) [2–3]. Anti-inflammatory and anti-oedematous activities following oral administration have also been reported, both in rodents [4] and in human patients with proven chronic venous insufficiency [5]. The anti-inflammatory properties are associated with down-regulation of pro-inflammatory mediators in vivo. Aescin helps decreasing the mortality resulting from the pathophysiology of sepsis in mice [6]. Moreover, it was proved to possess remarkable efficiency in both prevention and treatment of vascular disorders. Aescin increases the vascular tone by enhancing the formation of prostaglandin F2α in a variety of human tissues, including veins, and by allowing improved response to Ca^2+^ ions [1]. Aescin was also tested as a cytotoxic agent. It demonstrated trypanocidal activity in vitro and increased the longevity of *Trypanosome evansi* infected mice, however without curative effect [7]. In vitro incubation with cells of the C6 (glioma) and A549 (lung adenocarcinoma) tumoural lines showed that aescin has potent dose- and time-dependent antiproliferative effects [8]. Studies with human castration-resistant prostate cancer, both in vitro, using the cell lines PC-3 and DU-145, and in vivo using xenograft mice, showed cytotoxic effects for aescin, through the induction of apoptosis and G2/M cell cycle arrest [9].

Currently, aescin is mostly employed for venotonic action, being available in the form of topical formulations such as lotions, gels and creams. Many of these products resource to controlled release strategies, which can be achieved by encapsulating aescin into liposomes [2–3], phytosomes (phospholipidic self-emulsifying particles) [10], zeolites [11], poly(lactic co-glycolic acid) nanoparticles [12] or cyclodextrins. Cyclodextrins are cyclic oligosaccharides, typically with six (α-CD), seven (β-CD) and eight (γ-CD) units of α-D-glucose, which occur in nature from bacterial degradation of starch [13]. Their particular shape – a truncated cone with a hydrophobic cavity and a large number of hydroxy groups at the rims – enables them to include a variety of hydrophobic molecules and to enhance their stability in solution and their aqueous solubility. The only requisite of the encapsulated molecule, named guest, is that it possesses an adequate size and geometry to fit inside the cavity of the CD [14]. The ability of γ-CD to interact with triterpenic glycosides is known for over two decades, being first reported as a sequestering agent and sweetness inhibitor for strognin, a natural sweetener found in the Malaysian plant *Staurogyne merguensi* [15]. With glycemnic acid, a sweetness suppressing agent, γ-CD also acts as inhibitor, thus restoring one’s ability to taste sweets [16]. Inclusion of triterpenic compounds of medicinal interest is a topic of growing interest due to the ability of cyclodextrins to increase the solubility of these molecules. Currently, CDs are being used to afford non-toxic aqueous formulations with antitumoural single triterpenoids such as oleanoic [17–19], ursolic [18–19] and betulinic [18,20–22] acids. Ammonium glycyrrhizinate or glyciram (from licorice roots), sold in Russia and Japan as a natural anti-inflammatory, antiallergic and anti-spasmodic medicine, was also reported to become more stable against epoxidation upon inclusion into β-CD, thus gaining increased shelf-life [23–24].

Interaction of aescin with β-CD was studied only from an application viewpoint. A viscose textile grafted with β-CD allows loading a good amount of aescin for a sustained topical delivery [25]. The cosmeto-textile can be used to produce stocking with both compression and venotonic actions, with strong advantages for the symptomatic treatment of leg chronic venous insufficiency.

The aim of the present study is to evaluate the physicochemical aspects of the interaction of aescin with β-CD and γ-CD. The affinity and geometry of inclusion of aescin in the cavity of β-CD, having an inner diameter of 0.78 nm, is not known. Furthermore, and in spite of the many reports pointing to the preference of triterpenic guests for the larger host γ-CD [15–16,18,20], with cavity diameter of 0.95 nm, the γ-CD·aescin inclusion complex, herein reported, had not been studied to date. The present work demonstrates the stronger affinity of aescin to γ-CD and the formation of a 1:1 inclusion complex, presenting its most plausible geometry.

## Results and Discussion

### NMR studies in aqueous solution

#### Evaluation of the inclusion stoichiometries by the continuous variation method

**Figure 2:**
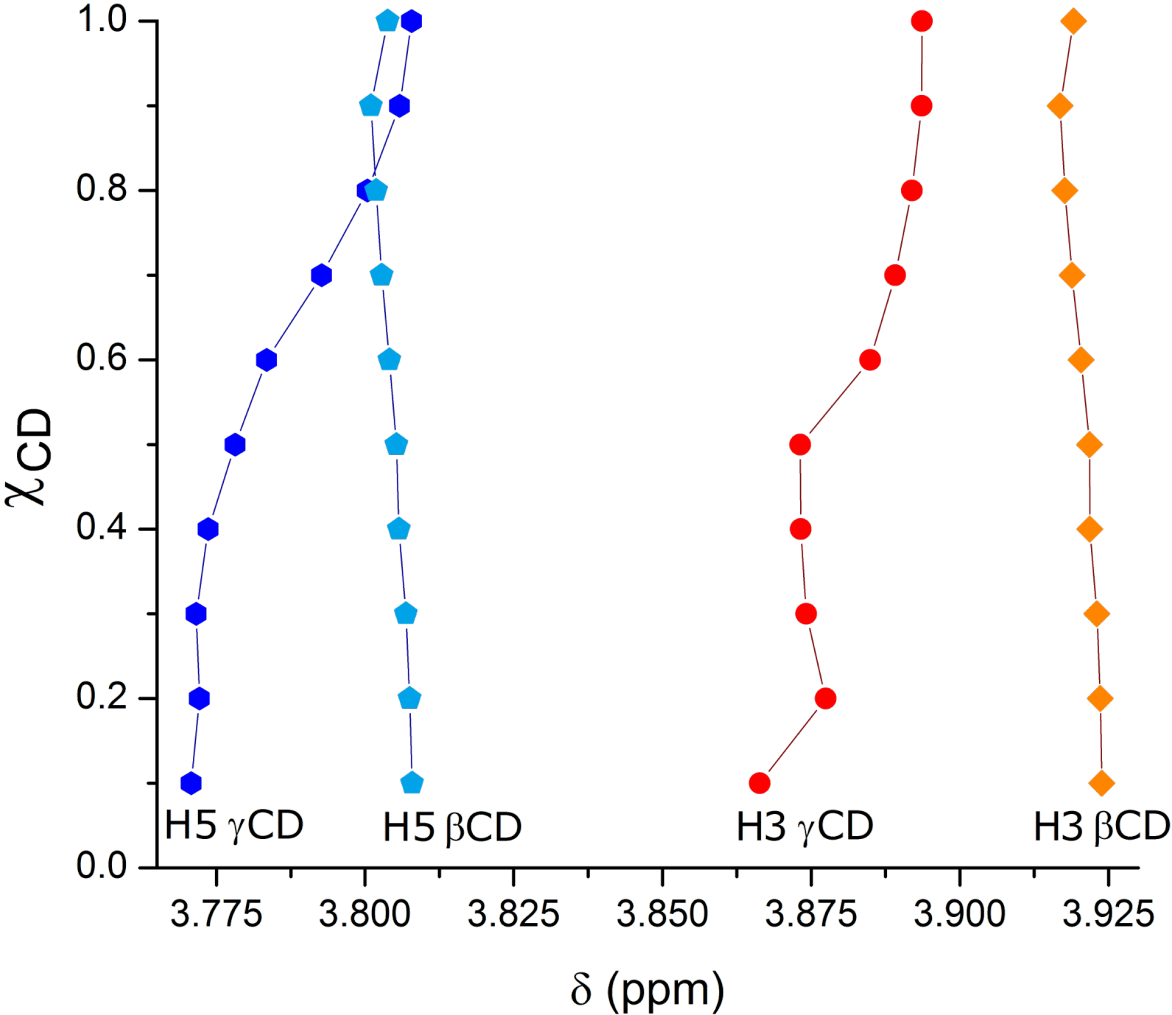
Chemical shift variation for the H3 and H5 protons of β-CD and γ-CD while performing the continuous variation method: [CD] + [aescin] = 10 mM. See Figure 9 for ^1^H labelling. Full lines connecting the experimental points are for indicative purposes only.

In the evaluation of the interaction of cyclodextrin with a guest molecule in aqueous solution, one first needs to investigate the stoichiometry [26]. For this, the continuous variation method (Job plot) [27] was employed. This assay consists in mixing aqueous solutions of host and guest in varying molar fractions while keeping constant the sum of their concentrations (see details in the dedicated Experimental section) and registering the variations in the chemical shifts of their protons (Δδ) relative to those of the individual components. The plot maximum corresponds to the preferred stoichiometric proportion of CD to aescin for each inclusion compound under study [27]. The H3 and H5 of the CDs are located inside their cavity and thus they are excellent probes for inclusion. Typically, these protons are the ones that exhibit the highest chemical shifts upon changes in the cavity's environment. The variation in the chemical shift of H3 and H5 of the two CDs under study as a function of the molar fraction of the CD/aescin mixtures is represented in the Figure 2.

**Figure 3:**
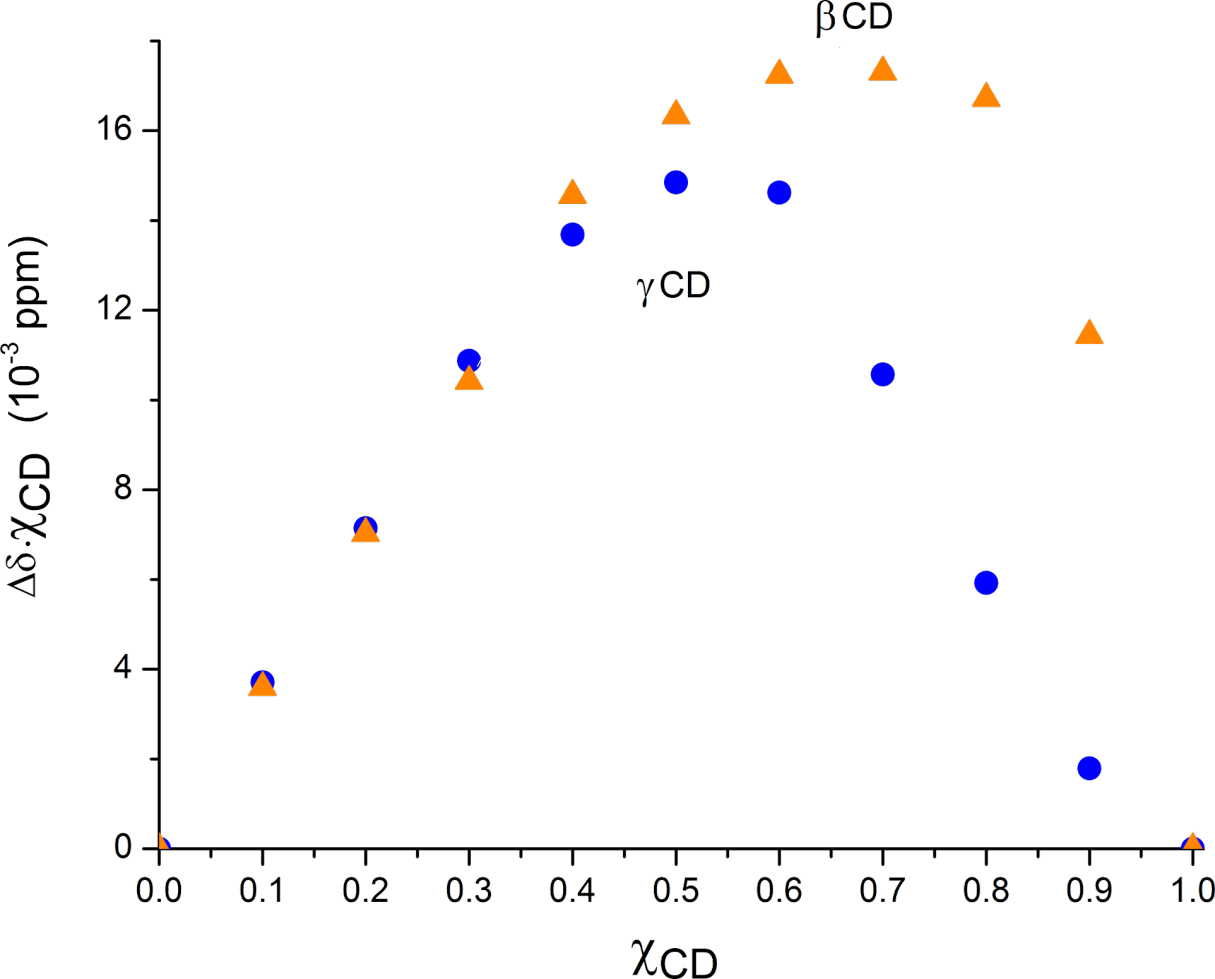
Job plot for the aescin complexes with β-CD (orange triangles) and γ-CD (blue dots) in D_2_O.

A closer look at Figure 2 allows observing that the variations in the H3 proton of γ-CD are not changing monotonically. For this reason, we have chosen to exclude the H3 shifts from the Job plot (an example for one of these data sets is shown in Supporting Information File 1, Figure S1.1). The shifts of protons H5 of both CDs were selected for the Job plot, depicted in Figure 3.

In the β-CD/aescin mixtures, the points associated with solutions having molar fractions of β-CD of 0.6 and 0.7 show approximately the same *y*-value. This indicates that the plot maximum is located between them, most likely at a molar fraction of 0.66 corresponding to a preferred stoichiometry of 2:1 (β-CD/aescin). In the γ-CD/aescin mixtures, the plot maximum was achieved at a molar fraction of γ-CD of 0.5, indicating that the preferred stoichiometry for the inclusion complex is 1:1; nonetheless, it should be noted that this plot is quite asymmetric and the shift observed at 0.6 (χ of γ-CD) denotes the presence of at least another specimen in solution, most likely the 2:1 γ-CD/aescin species.

#### Apparent association constant

The samples used for the calculation of the apparent association constant (*K*_app_) comprised aqueous solutions with a fixed concentration of cyclodextrin and variable concentrations of aescin (in excess from five to ten folds regarding the concentration of the host, see the Experimental section for further details). The inclusion constants of the complexes were estimated according to a graphical method developed by Seal et al. [28] by rearrangement of the Benesi–Hildebrand equation [29]. This method afforded values of 894 M^−1^ (±13.5%) for γ-CD·aescin and 715 M^−1^ (±10.6%) for 2β-CD·aescin, where values between parenthesis indicate the relative standard deviation. This may indicate a preference of aescin towards the larger cavity of γ-CD. It must be noted, though, that the possible occurrence of more than one species in solution, as denoted by the Job plot, implies that the *K*_app_ values herein presented are only a rough estimate of affinity. Furthermore, the values are within the same order of magnitude, meaning that the affinity of aescin towards these two hosts is not markedly different.

#### ROESY spectrum of γ-CD·aescin

**Figure 4:**
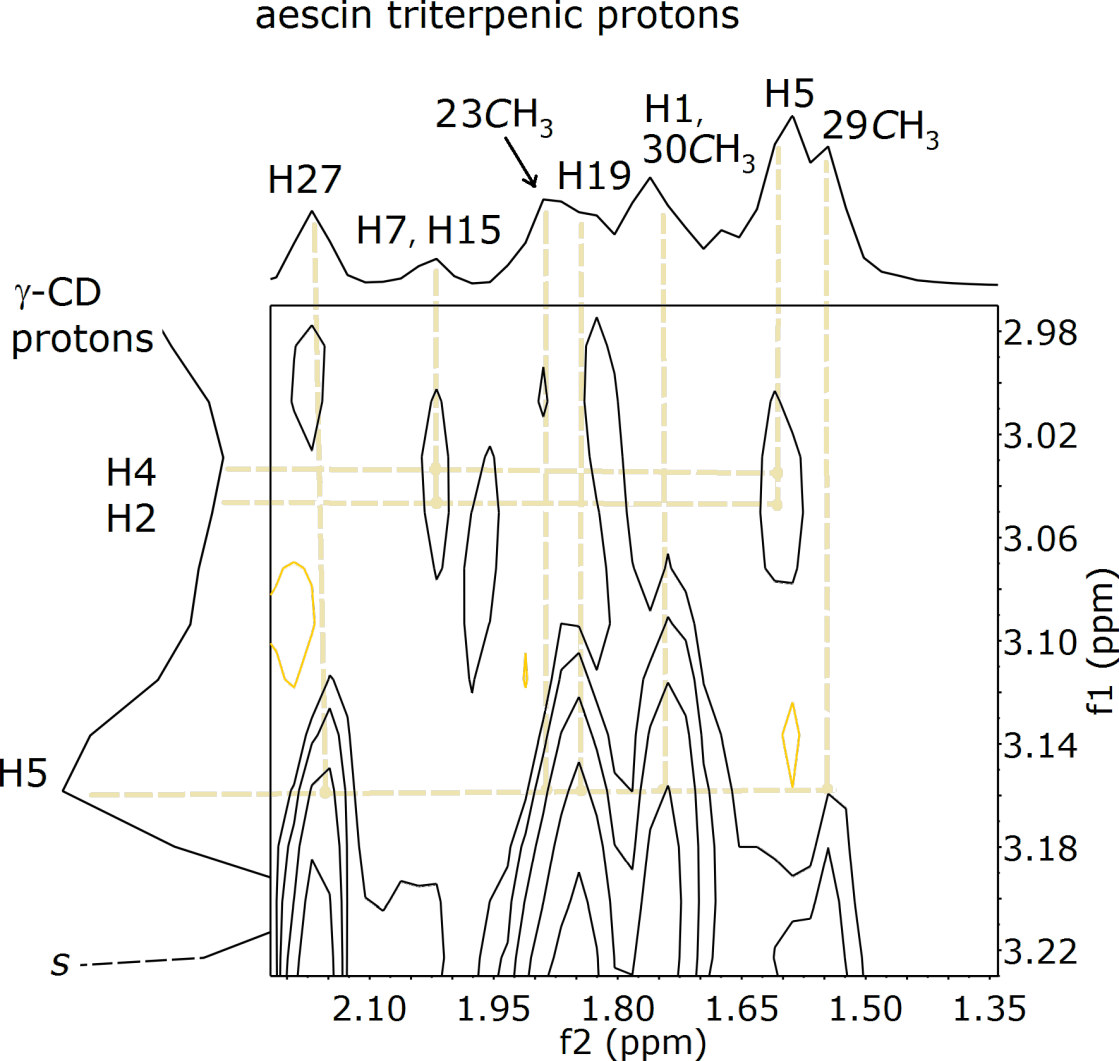
Partial 2D ROESY spectrum of a water/methanol solution containing equimolar amounts of γ-CD and aescin.

**Figure 5:**
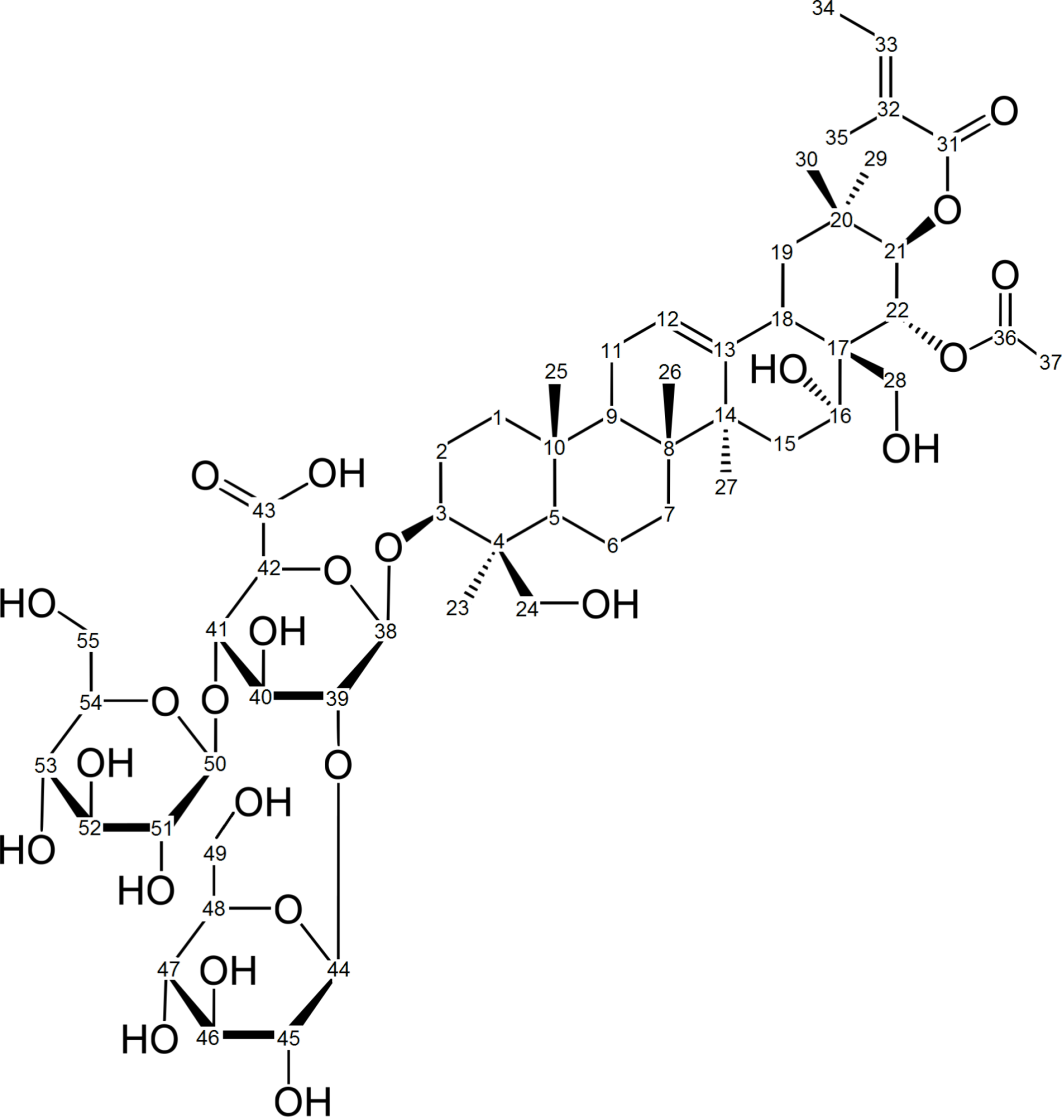
Carbon (and corresponding proton) numbering of aescin.

The 2D ROESY spectrum of a D_2_O/CD_3_OD solution of an aescin and γ-CD mixture is consistent with inclusion. An expansion showing the region of interest to the observation of host–guest interactions is depicted in the Figure 4 (refer to the Supporting Information File 2 for the full spectrum). The resonance of the H5 protons, which are directed towards the inner cavity of γ-CD, appears centred at 3.15 ppm, slightly shifted regarding the values reported for pure γ-CD [30]. This feature is associated with the formation of inclusion complexes in solution. Furthermore, correlations were found between the host’s H5 and several protons ascribed to the triterpenic moiety of aescin and some of its methyl groups, namely H1, H19, H27 and methylene protons H23, H29 and H30 (see Figure 5 for labelling) [31]. In the region associated with protons H2 and H4 of γ-CD, some correlations with aescin triterpenic protons were also found, namely with H5, H7 and H15. This suggests that aescin is included into γ-CD by its triterpenic moiety, in a good match with the geometry calculated for the complex γ1 in the following section.

### Investigation of the CD·aescin inclusion modes by DFT calculations

The geometry of interaction of aescin with β-CD and γ-CD was further studied by means of theoretical calculations. For this, and given that aescin is a mixture of components, the main active ingredient, aescin Ib, was chosen as representative guest molecule. Its interaction with the CDs was evaluated by means of DFT calculations by creating different complex geometries that could represent the real complex. The geometries were optimised at the M06-2X/6-31g(d) level with tight convergence criteria, including dispersion energy corrections (D3) and in the presence of a solvation model to simulate the presence of water. It should be mentioned that all atoms were optimised at the same level of theory, i.e., the ONIOM approach was not considered. Afterwards NMR chemical shifts were estimated at the more accurate M06-2X/6-311g(d,p) level with D3 correction and the solvation model, while using the GIAO algorithm. Furthermore, for each optimised inclusion complex geometry, the ^1^H NMR chemical shifts of signals of the protons H3 and H5 of the host were calculated and compared with those calculated for the neat cyclodextrins.

**Figure 6:**
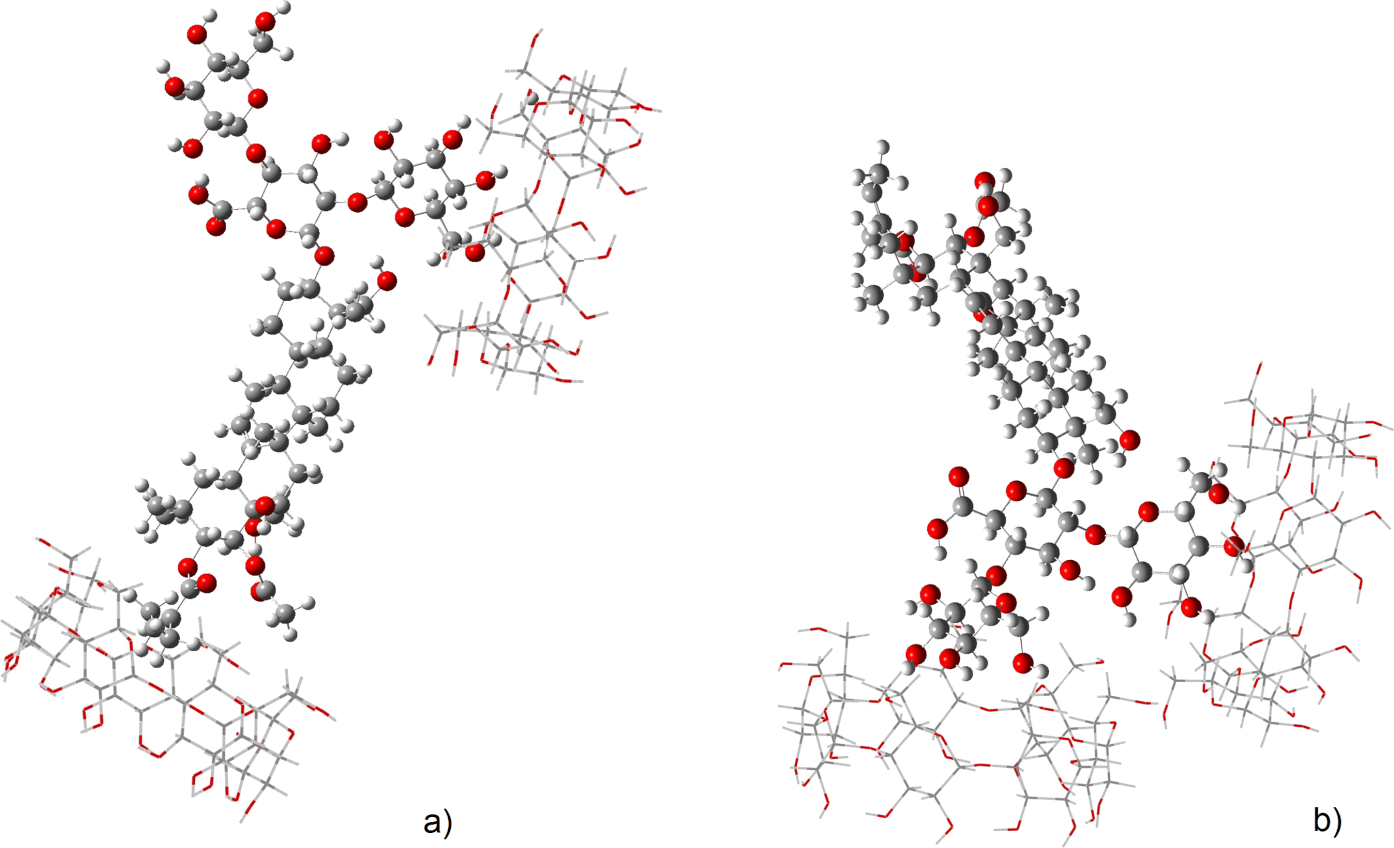
Optimised structures of the theoretical 2β-CD·aescin complex β1 (a) and complex β2 (b).

For the 2β-CD·aescin complex two possible geometries were optimised. These structures are represented in the Figure 6 and will be hereafter named complex β1 and complex β2.

The first observation that strikes to the sight is that the cavity of β-CD is not large enough to allow a deep encapsulation of the guest molecule. This was reflected by the calculated NMR chemical shifts, which yielded negligible changes matching the experimental observation for H3 and H5 (Figure 2). In terms of formation energies of the theoretical 2β-CD·aescin structures, the complex β2 is the most stable, while the complex β1 is less stable by 135 kJ/mol. From Figure 6, it can be seen that the structure of complex β2 shows vicinal β-CD units resulting in an extended network of H-bonds which does not occur in the complex β1, being that a reason for the lower energy of the former structure.

**Figure 7:**
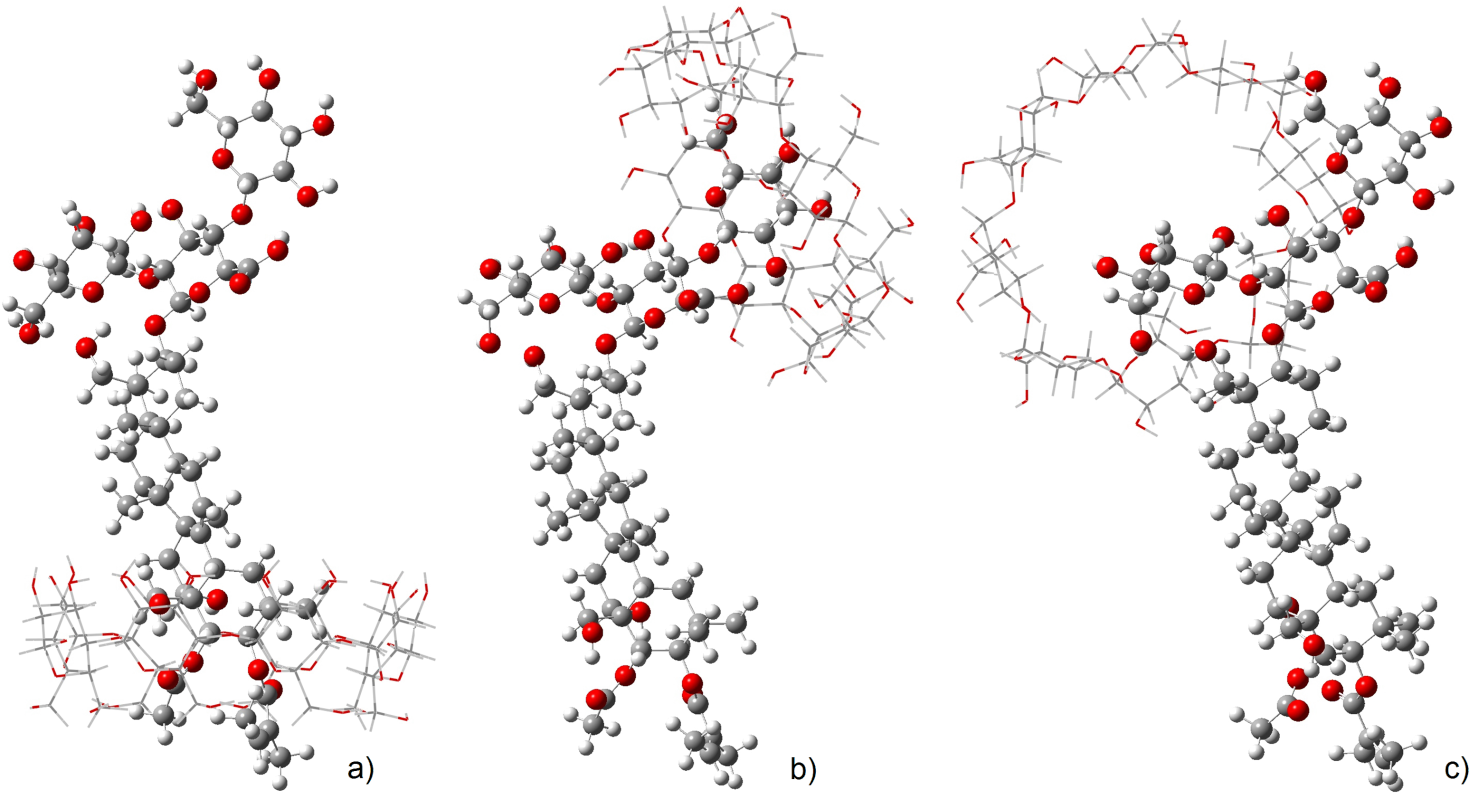
Optimised geometries for three different hypothetical structures of γ-CD·aescin, deemed complex γ1 (a) complex γ2 (b) and complex γ3 (c). The structures have the same orientation to facilitate viewing.

For the γ-CD·aescin complex, three possible geometries were computed. The resulting optimised structures, shown in Figure 7, allow observing that the main interactions in the γ-CD·aescin complexes γ1 and γ2 occur for an almost complete encapsulation while for complex γ3 the interaction occurs at the upper rim of γ-CD.

**Figure 8:**
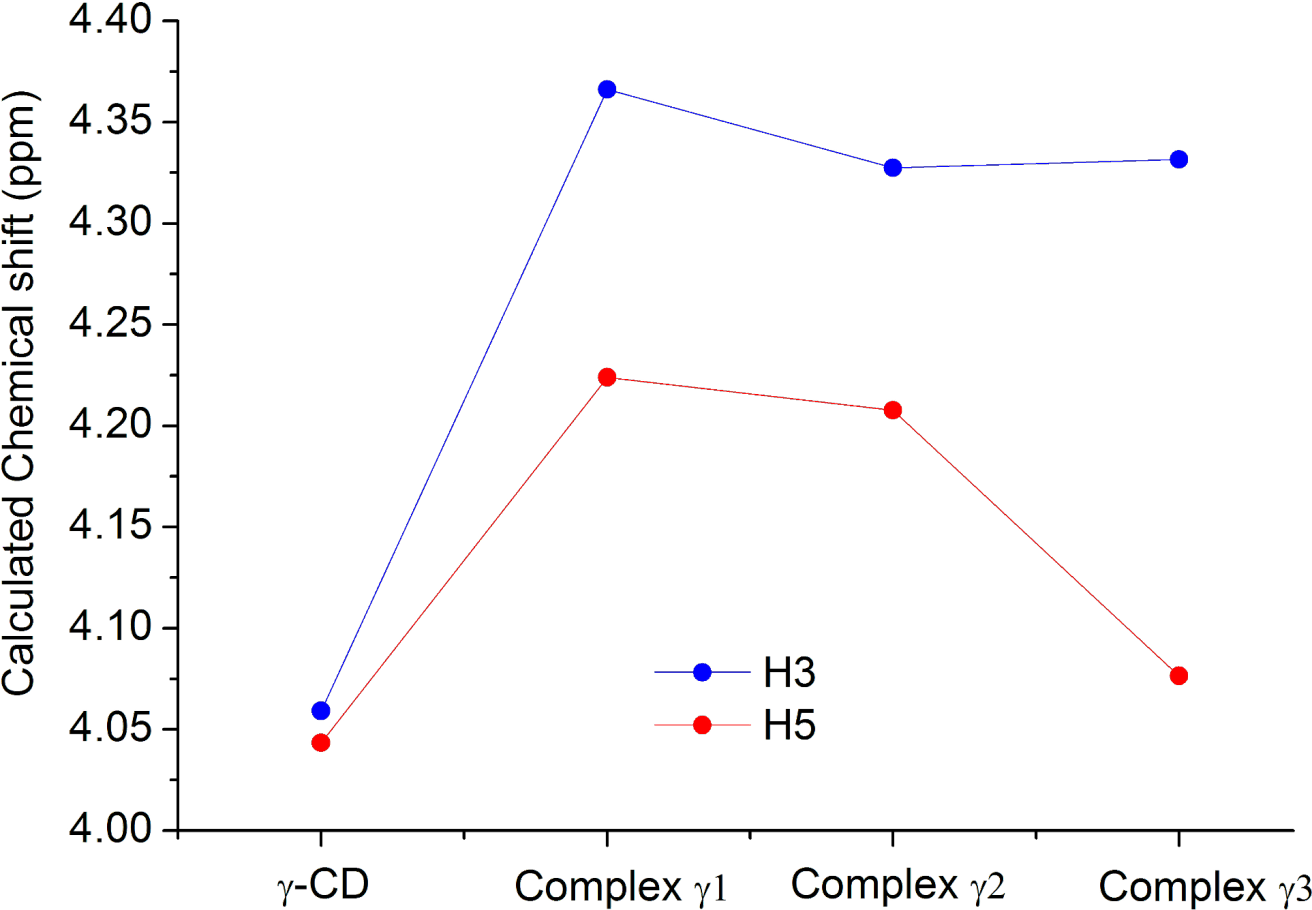
DFT [M06-2X/6-311G(d,p) with D3 dispersion and IEFPCM polarizable continuum solvent corrections] calculated NMR chemical shifts for protons H3 and H5 for neat γ-CD and the γ-CD·aescin theoretical complexes γ1, γ2 and γ3.

The formation energies of the structures represented in the Figure 7 follow the order complex γ1 ≤ complex γ2 << complex γ3. The most stable geometry is found with complex γ1 and it involves inclusion of the triterpenic moiety of aescin into γ-CD. Nonetheless, the difference in energy to that of complex γ2 is only +27.1 kJ/mol. This means that this second geometry, in which γ-CD docks at one of the glucose residues, is also quite plausible. Finally, complex γ3 has the less stable geometry, with an energy difference of +82.7 kJ/mol. These findings are confirmed by the calculated NMR chemical shifts (Figure 8), which show changes in the two most stable structures, while the third displays little changes as will be discussed next.

As can be seen from Figure 8, the calculated values match the same trend as the experimental ones for neat γ-CD. The same applies to the observed changes in the chemical shifts after formation of the complexes. Comparing the calculated and experimental data (for the Job plot) complex γ3 can be discarded, since it does not allow for a change in the chemical shift of H5. This result further confirms that the most plausible geometries are those of complexes γ1 and γ2. It should be mentioned as well that the predicted shifts from neat to complexed in γ-CD for H3 and H5 are in excellent agreement with the experimentally measured NMR chemical shifts for those nuclei where the shift experienced for H5 is larger than that for H3.

## Conclusion

In the present work, the interaction of aescin with the oligosaccharide hosts β-CD and γ-CD is investigated, combining NMR characterisation in aqueous solution with DFT theoretical calculations to determine the stoichiometry, the host–guest affinity, and the geometry of inclusion. The results show that aescin forms preferentially 2:1 complexes with β-CD, having a moderate affinity (*K*_app_ = 715 M^−1^) for this host. DFT calculations allowed postulating that the two β-CD molecules interact with aescin at one of the glucose fragments to form a complex stabilised by an extensive network of hydrogen bonds. Inclusion of aescin into γ-CD is favoured by the larger diameter of its cavity, where aescin fits more adequately. This results in the occurrence of complexes with 1:1 stoichiometry and a slightly higher host−guest affinity (*K*_app_ = 894 M^−1^). Regarding the geometry of inclusion of the γ-CD·aescin complex, ROESY indicates inclusion of the triterpenic moiety, with correlations between the host cavity protons (H5) and several triterpenic protons of aescin. DFT calculations further confirmed that this geometry is the most stable. The γ-CD·aescin inclusion complex may find applications in liquid formulations. Aescin solutions produce foam when shaken, which causes an undesirable visual effect (refer to the Supplementary Information File 4 for details). γ-CD stabilises aescin and allows to shorten the time of permanence of the foam bubbles in solution.

## Experimental

### Materials

Aescin (purum, mixture of saponins, ≥96.0%) was obtained from Fluka and used as received. Pharmaceutical grade β-CD and γ-CD (manufactured by Wacker, tradenames Cavamax W7 and W8, water content of ca. 14% for β-CD and 9% for γ-CD) were kindly donated by Ashland Specialty Ingredients (Düsseldorf, Germany). All other materials and solvents were of analytical reagent grade.

### NMR spectroscopy

One- and two-dimensional NMR spectra were recorded on a Bruker Avance 500 spectrometer at 500.13 MHz at room temperature. ^1^H NMR spectra recorded in D_2_O were referenced to HOD at δ = 4.79. For the Rotating-frame Overhauser Spectroscopy (ROESY, spin lock 200 ms) experiments, solutions with equimolar quantities of aescin and γ-CD were used (concentration 15 μM, solvent D_2_O/CD_3_OD in an 80:20 proportion).

### Job plot

**Figure 9:**
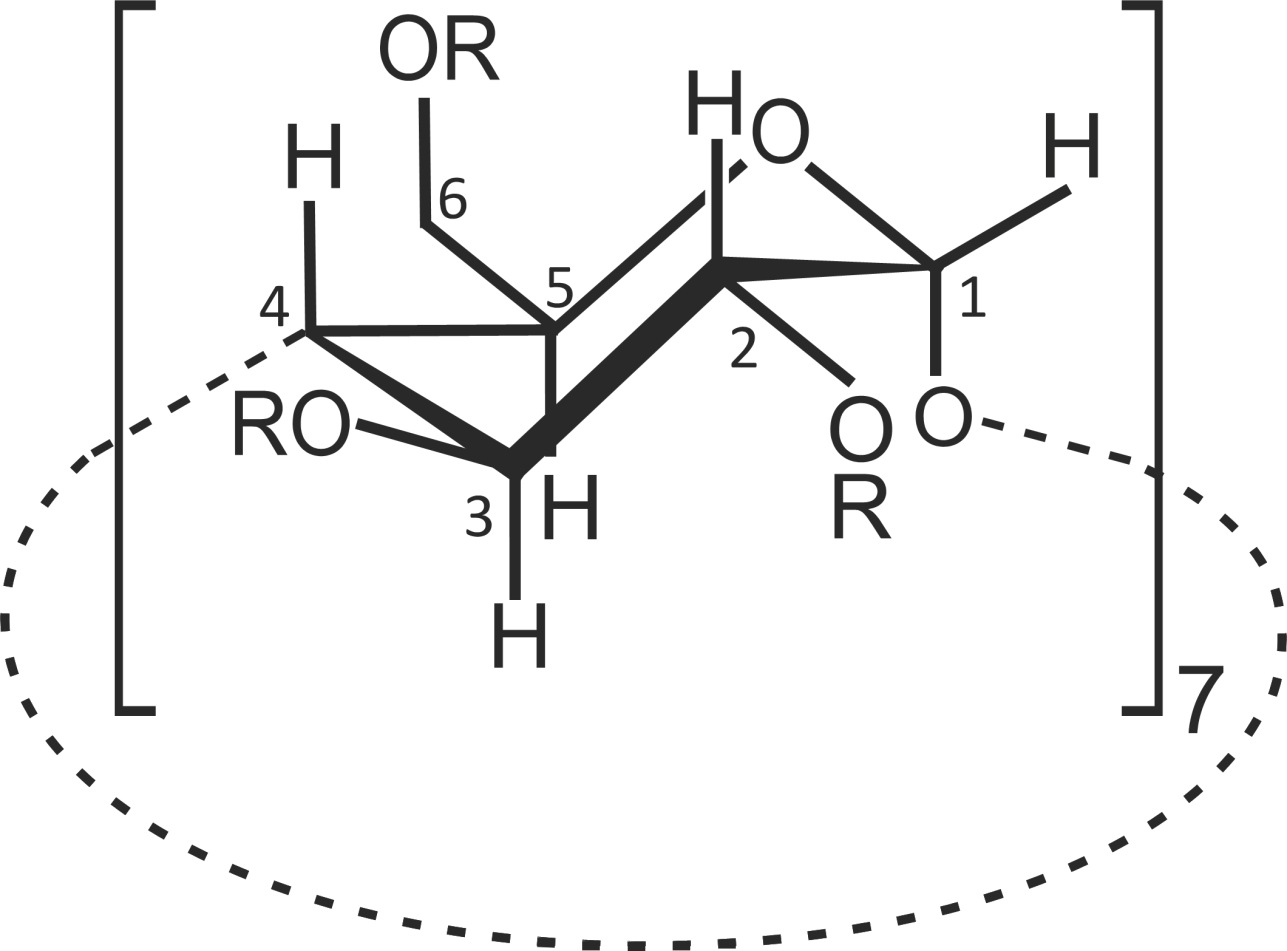
Carbon (and corresponding proton) numbering of cyclodextrins, herein demonstrated for β-CD.

The stoichiometry of CD·aescin complexes in deuterium oxide solution was determined using the continuous variation method or Job's method [27]. It involves running a series of experiments varying the host and guest concentrations while keeping their sum constant ([CD] + [aescin]) at well-defined *r*-values (*r* = [CD]/{[CD] + [aescin]}). In particular, 10 mM fresh D_2_O solutions of aescin and each CD were mixed (i) to constant volume, i.e., the sum of the initial concentrations of β-CD and aescin remained equal to 10 mM, and (ii) to defined values of *r*, where *r* took values from 1/10 to 9/10, in steps of 1/10. The stoichiometry was finally determined by plotting Δδ·[CD] against *r*, where Δδ is the NMR shift of the selected proton, H5 (see Figure 9 for atom numbering), and finding the *r* value corresponding to the maximum of this distribution.

### Determination of the apparent formation constant (*K*_app_)

The equilibrium for the inclusion process in aqueous solution is ruled by a constant deemed apparent association constant or *K*_app_. The inclusion constant was estimated by an adaptation of the Benesi–Hildebrand method [29], derived by Seal et al. [28], as shown in Equation 1:

[1]
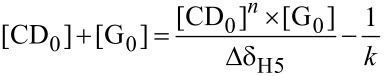


In complexes having 1:1 stoichiometry, *n* = 1 and in complexes with 2:1 stoichiometry, as is the case of β-CD with aescin, *n* = 2. For each host–guest system, five NMR samples were prepared, each containing [CD] = 0.25 mM and [aescin] = 2.5 to 4.5 mM. Plotting ([CD_0_] + [G_0_]) vs ([CD_0_]*^n^* + [G_0_])/Δδ_H5_ allows obtaining 1/*K*_app_ as the yy-intercept (see Supporting Information File 1 for the plot graphics).

### Computational details

DFT calculations [32] were performed using the Gaussian09 program, revision D01 [33], with the M06-2X hybrid functional, which includes a mixture of 54% Hartree–Fock exchange with DFT exchange correlation as developed by Truhlar and Zhao [34]. Pople’s 6-31G(d) basis set was used on all atoms. All geometries were optimised without any geometry constraints at the M06-2X/6-31G(d) level of theory as mentioned above. The optimisation approach also included the use of tight convergence criteria. To evaluate the effects of solvation, to account for the effects of water the IEFPCM model was used for the description of the solvent continuum. Furthermore, the ONIOM approach was not used with all atoms being optimised at the same level of theory.

**Figure 10:**
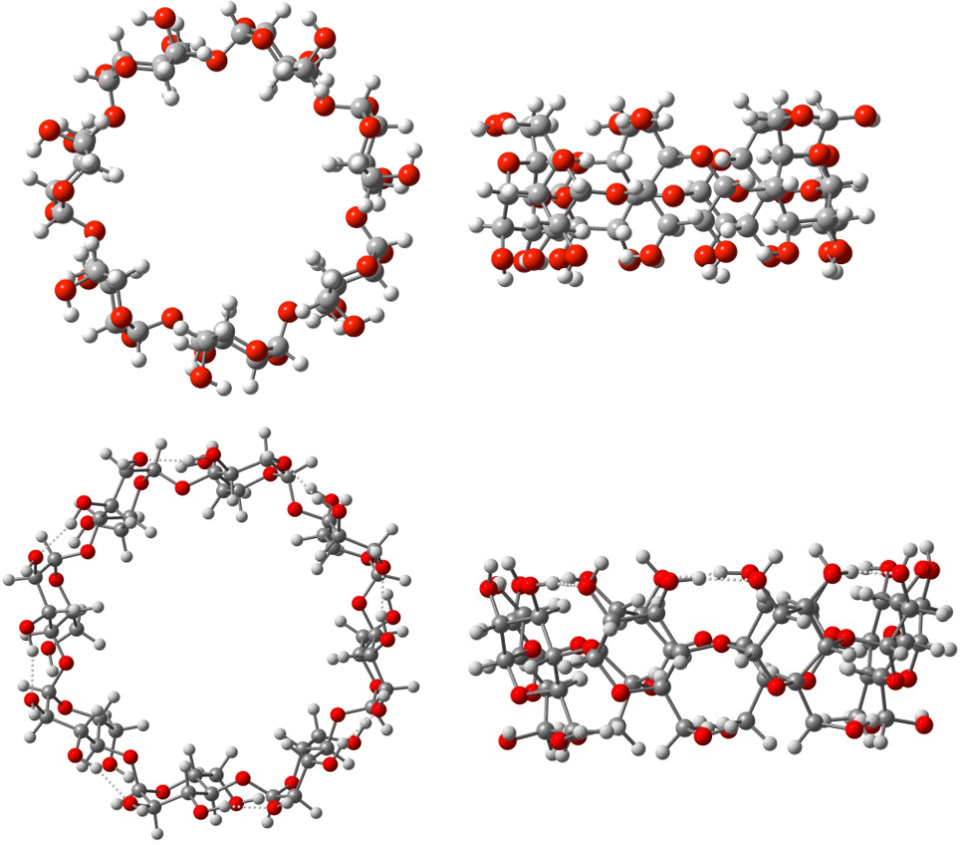
Optimised geometry for β-CD (top) and γ-CD (bottom), viewed from above (left) and from the side (right).

The initial geometries of the hosts and the guest were taken from structures available in the Cambridge Structural Database (CSD) [35]. For β-CD, the coordinates were procured from the structure of inclusion complex β-cyclodextrin·*S*-(+)-ibuprofen chlathrate hydrate (refcode TUXKUS) [36]; for γ-CD data was taken from the inclusion complex γ-cyclodextrin·methanol·*n*·H_2_O (refcode NUNRIX [37]), in which the glucopyranose units already feature a high symmetry due to the presence of the methanol guest. The geometry of γ-CD after further optimisation is depicted in Figure 10. The initial geometry of aescin Ib was taken from its single-crystal diffraction data (refcode GACZIT [38]). A similar simulation strategy has been adopted by some of us previously to address accurate geometries and energies of organic molecules [39].

NMR shielding tensors were calculated using the gauge-independent atomic orbital method (GIAO) [40–44] with the same functional, dispersion correction and solvent continuum model, an improved basis set – 6-311G(d,p) – on the geometries optimised previously at the M06-2X/6-31g(d) level.

For comparison purposes the same calculations were accomplished with both the M06-2X and the B3LYP hybrid functional, but without the D3 or solvation corrections. In all cases the obtained results were consistent (including with the M06-2X with D3 and solvation corrections).

## Supporting Information

The details on the host–guest association constant (*K*_app_) estimation by the graphical method of Seal et al. [28] are demonstrated, with one example for each cyclodextrin, in Supporting Information File 1, as well as the plot of the H3 shifts of γ-CD according to the Job method.

The selected region of the ROESY spectrum of γ-CD·aescin is shown in Supporting Information File 2.

The γ-CD·aescin complex formation in the solid state was also investigated and characterised by FTIR spectroscopy. Results are presented in Supporting Information File 3.

The foam disrupting capacity of the encapsulated aescin is presented in Supporting Information File 4.

File 1 Supplement to ^1^H NMR studies in solution.

File 2 ROESY spectrum of γ-CD·aescin.

File 3 FTIR studies of the solid γ-CD·aescin inclusion compound.

File 4 Studies on the influence of encapsulation on the foam-forming properties of aescin.
