# Supplementary material for: Association of aescin with β- and γ-cyclodextrins studied by DFT calculations and spectroscopic methods
Source: Beilstein J Nanotechnol. 2017 Feb 3;8:348–57. doi: 10.3762/bjnano.8.37 (PMC5566205; doi:10.3762/bjnano.8.37)
Supplement: File 1 — Supplement to 1H NMR studies in solution. [file Beilstein_J_Nanotechnol-08-348-s001.pdf]

Supporting Information 1

for

Association of aescin with β- and γ-cyclodextrins studied by DFT calculations and spectroscopic methods

Ana I. Ramos*[1,2], Pedro D. Vaz[3,4], Susana S. Braga[5] and Artur M. S. Silva[5]

Address: [1]CICECO, Complexo de Laboratórios Tecnológicos, Campus Universitário de Santiago, 3810-193 Aveiro, Portugal; [2]Current affiliation: INEGI-FEUP Faculty of Engineering of the University of Porto, Rua Dr. Roberto Frias, 4200-465, Porto, Portugal; [3]CQB, Departamento de Química e Bioquímica, Faculdade de Ciências da Universidade de Lisboa, 1749-016 Lisboa, Portugal; [4]ISIS Neutron & Muon Source, Rutherford Appleton Laboratory, Chilton, Didcot, Oxfordshire OX11 0QX, United Kingdom and [5]QOPNA, Departamento de Química, Universidade de Aveiro, Campus de Santiago, 3810-193 Aveiro, Portugal

Email: Ana I. Ramos - shortinha.sa@gmail.com

* Corresponding author

Supplement to ^{1}H NMR studies in solution

a) Attemptive Job plot for the H3 proton of γCD in γCD·aescin mixtures

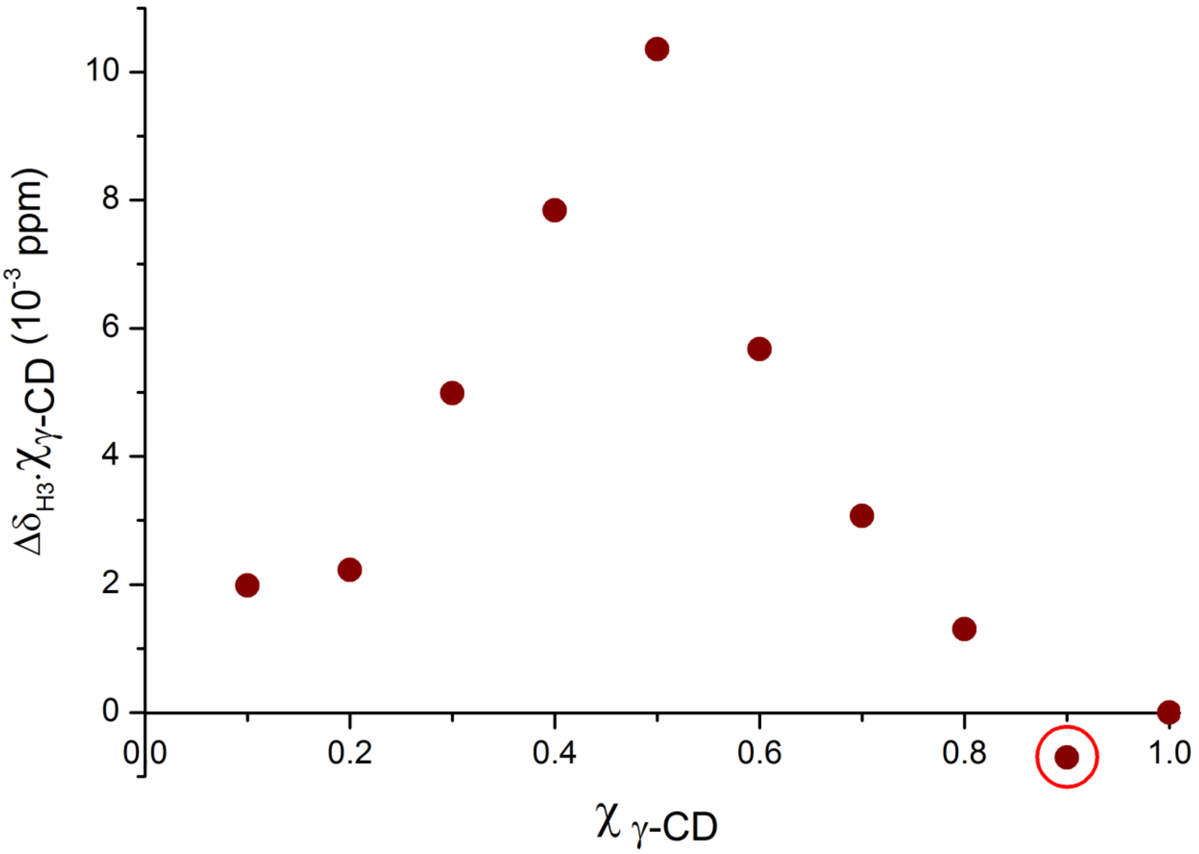

Figure S1.1: Job plot for the H3 proton of γ-CD in aqueous solutions containing γ-CD and aescin in a gradient of molar fraction. Note how the inconsistency in the proton shifts affords a negative value for the data point corresponding to the molar fraction of 0.9 (circled with red).

b) Apparent Inclusion Constants for βCD·aescin and γCD·aescin

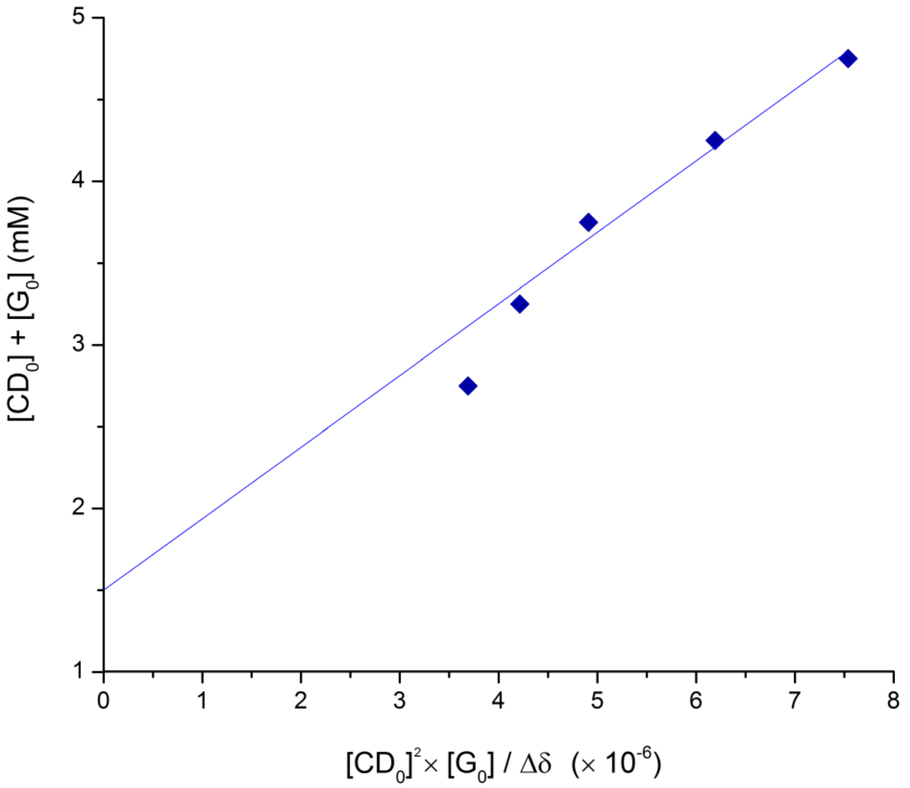

Figure S1.2: Graphical analysis of ^{1}H NMR shifts of the H5 proton of β-CD of one assay comprising a series of aqueous solutions containing 0.25 mM of β-CD and 2.5, 3.0, 3.5, 4.0 and 4.5 mM of aescin. Data is fitted to a line with r^2 value of 0.972 and x_0 (yy-intercept) of 0.00151, which allows estimating, for this data set, $K_{app} = 1/x_0 = 662\ M^{-1}$ (the values presented in the manuscript are the average (± RSD) of different calculated K_{app} values). The first data point was not used in the data fitting.

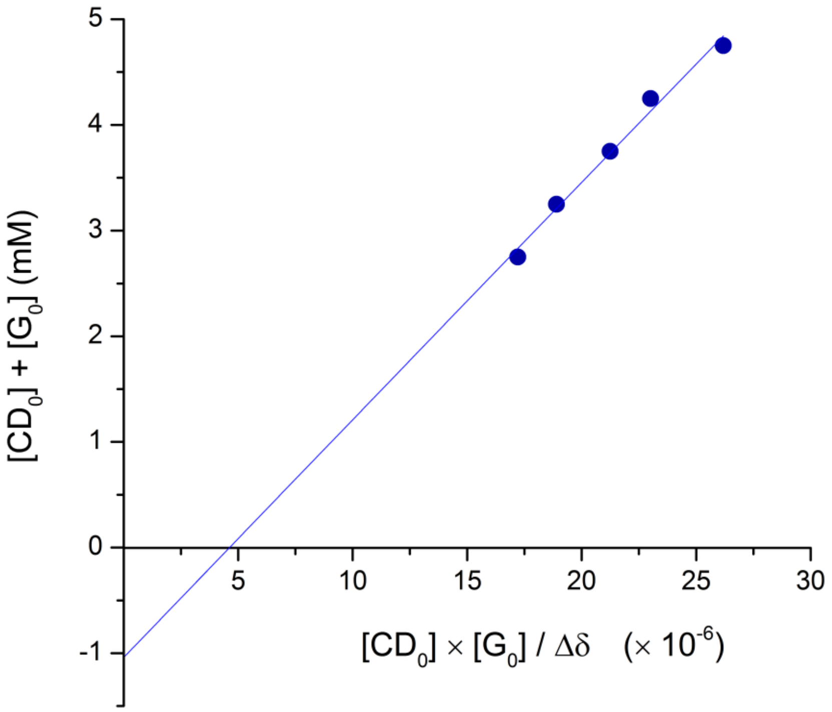

Figure S1.3: Graphical analysis of ^{1}H NMR shifts of the H5 proton of γ-CD of one assay comprising a series of aqueous solutions containing 0.25 mM of γ-CD and 2.5, 3.0, 3.5, 4.0 and 4.5 mM of aescin. Data is fitted to a line with r^2 value of 0.9834 and x_0 (yy-intercept) of -0.00102, which allows estimating $K_{app} = 1/x_0 = 980\ M^{-1}$.
