# Supplementary material for: Association of aescin with β- and γ-cyclodextrins studied by DFT calculations and spectroscopic methods
Source: Beilstein J Nanotechnol. 2017 Feb 3;8:348–57. doi: 10.3762/bjnano.8.37 (PMC5566205; doi:10.3762/bjnano.8.37)
Supplement: File 2 — ROESY spectrum of γ-CD·aescin. [file Beilstein_J_Nanotechnol-08-348-s002.pdf]

Supporting Information 2

for

Association of aescin with β- and γ-cyclodextrins studied by DFT calculations and spectroscopic methods

Ana I. Ramos*[1,2], Pedro D. Vaz[3,4], Susana S. Braga[5] and Artur M. S. Silva[5]

Address: [1]CICECO, Complexo de Laboratórios Tecnológicos, Campus Universitário de Santiago, 3810-193 Aveiro, Portugal; [2]Current affiliation: INEGI-FEUP Faculty of Engineering of the University of Porto, Rua Dr. Roberto Frias, 4200-465, Porto, Portugal; [3]CQB, Departamento de Química e Bioquímica, Faculdade de Ciências da Universidade de Lisboa, 1749-016 Lisboa, Portugal; [4]ISIS Neutron & Muon Source, Rutherford Appleton Laboratory, Chilton, Didcot, Oxfordshire OX11 0QX, United Kingdom and [5]QOPNA, Departamento de Química, Universidade de Aveiro, Campus de Santiago, 3810-193 Aveiro, Portugal

Email: Ana I. Ramos - shortinha.sa@gmail.com

* Corresponding author

ROESY spectrum of γ-CD·aescin

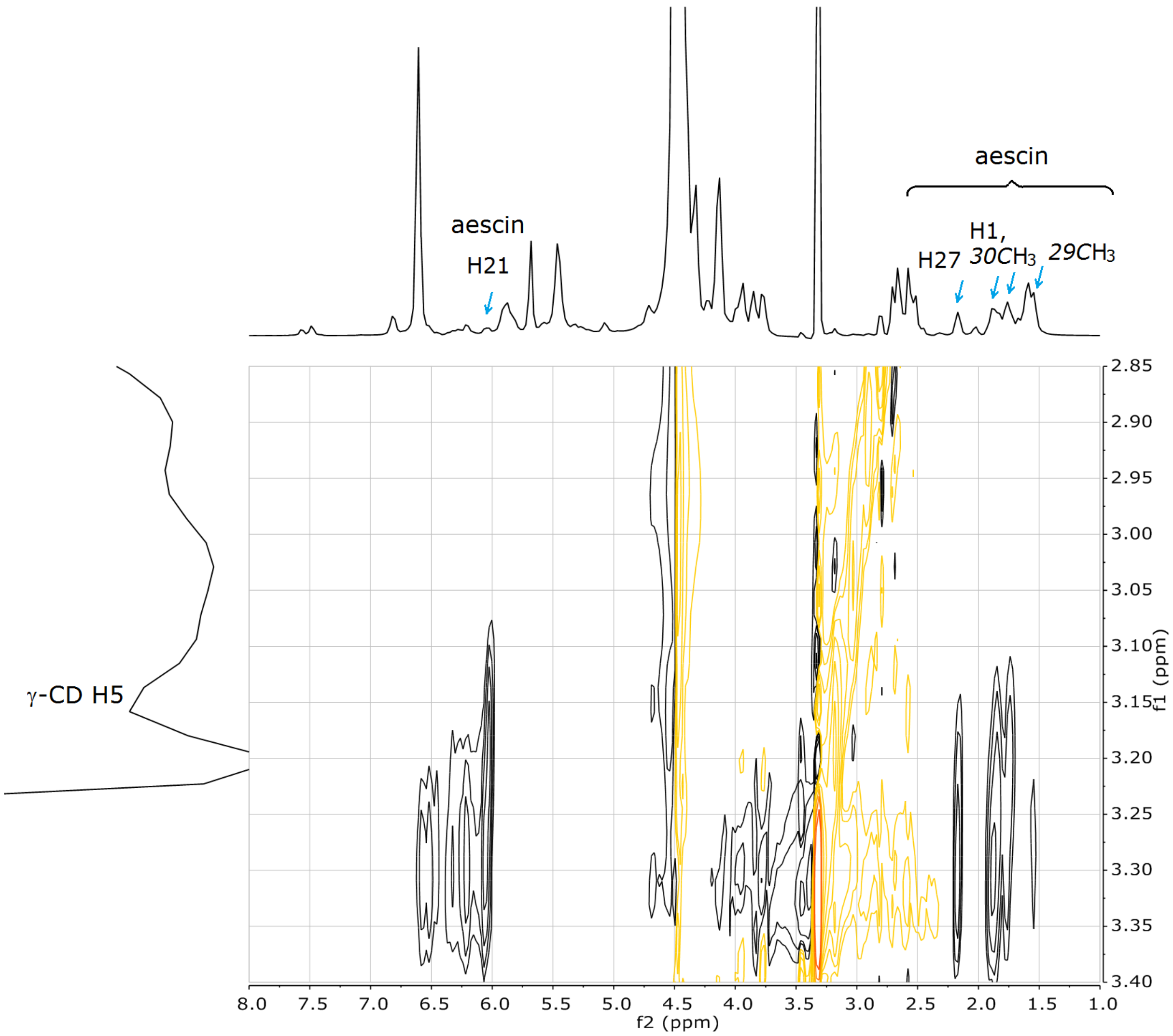

Figure S2.1: Selected region of the ROESY NMR spectrum of an equimolar γ-CD/aescin mixed solution in D_2O and CD_3OD, emphasising the interactions of the H5 proton of γ-CD with several triterpene protons of aescin. These protons of aescin are marked with blue arrows on the horizontal axis. The assignment of the aescin protons was done based on the report by Oledzka et al. [1].

References

1. Oledzka, E.; Pachowska, D.; Sobczak, M.; Lis-Cieplak, A.; Nalecz-Jawecki, G.; Zgadzaj, A.; Kolodziejski, W. *Polymers (Basel, Switz.)* **2015,** *7,* 1820–1836. doi:10.3390/polym7091484
