# Supplementary material for: Association of aescin with β- and γ-cyclodextrins studied by DFT calculations and spectroscopic methods
Source: Beilstein J Nanotechnol. 2017 Feb 3;8:348–57. doi: 10.3762/bjnano.8.37 (PMC5566205; doi:10.3762/bjnano.8.37)
Supplement: File 3 — FTIR studies of the solid γ-CD·aescin inclusion compound. [file Beilstein_J_Nanotechnol-08-348-s003.pdf]

Supporting Information 3

for

Association of aescin with β- and γ-cyclodextrins studied by DFT calculations and spectroscopic methods

Ana I. Ramos*[1,2], Pedro D. Vaz[3,4], Susana S. Braga[5] and Artur M. S. Silva[5]

Address: [1]CICECO, Complexo de Laboratórios Tecnológicos, Campus Universitário de Santiago, 3810-193 Aveiro, Portugal; [2]Current affiliation: INEGI-FEUP Faculty of Engineering of the University of Porto, Rua Dr. Roberto Frias, 4200-465, Porto, Portugal; [3]CQB, Departamento de Química e Bioquímica, Faculdade de Ciências da Universidade de Lisboa, 1749-016 Lisboa, Portugal; [4]ISIS Neutron & Muon Source, Rutherford Appleton Laboratory, Chilton, Didcot, Oxfordshire OX11 0QX, United Kingdom and [5]QOPNA, Departamento de Química, Universidade de Aveiro, Campus de Santiago, 3810-193 Aveiro, Portugal

Email: Ana I. Ramos - shortinha.sa@gmail.com

* Corresponding author

FTIR studies of the solid γ-CD·aescin inclusion compound

Experimental details

For the investigation of the interaction of aescin with γ-CD by means of FTIR spectroscopy, their 1:1 aqueous co-solution was frozen and freeze-dried. The resulting voluminous solid was co-ground with KBr (roughly 2 mg of sample for 100 mg of KBr) to produce solid pellets. Spectra of the samples in KBr were collected in a Mattson 7000 FTIR spectrometer (resolution 2.0 cm^{-1}; 128 scans per spectrum).

Results and discussion

The spectrum of γ-CD·aescin isolated as a solid product was collected and compared with those of γ-CD heptahydrate and aescin. Aescin presents a number of broad bands, as expected due to the fact that it is a complex product comprising different oscillators of the same kind. Upon inclusion into γ-CD, some bands were overlapped by those of the host, as there are numerous groups of the same chemical nature, and the ν (C=C) at 1647 cm^{-1} is overlapped by the δ (H–O–H) of the hydration water molecules of the host. Nonetheless, a few characteristic bands of aescin are still observed, and listed in Table S2.1.

Table S2.1: Selected FTIR bands for aescin and its inclusion compound with γ-CD.

Aescin	γ-CD·aescin	Approximate Description [a]
1735	1735	ν $(C=O)_{\text{COOH group}}$
1722	1718	ν (C=O) $_{\text{ester groups}}$
1377	1374	δ $(C–H)_{\text{alyphatic}}$
1265	1256	δ (C–OH)

[a] Description of the vibrational modes was made according to Khan, L.; Kifayatullah, Q.; Ahmad, K.D.; Arfan, M., Preparation of water soluble aescin from *Aesculus indica* seeds. *J. Chem. Soc. Pak*. 1994, 16(4), 269, and to Colthup, N. Introduction to Infrared and Raman Spectroscopy, 2nd Edition, 1975, Elsevier. ISBN: 978-0-12-182552-2

Most of the observed shifts are associated with vibrational modes of the terpenoid moiety, whereas, for instance, the carbonyl stretch of the glucopyranosiduronic acid has suffered no

shift upon inclusion into γ-CD. It is thus plausible to assume that, in the solid-state, inclusion involves mostly the penetration of the terpenoid fragment into the host cavity, as schematised below.

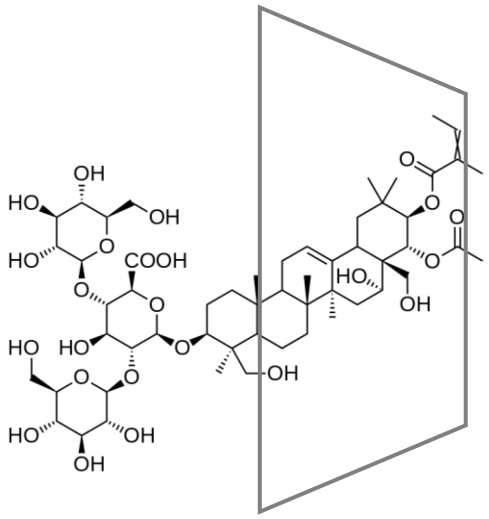

Figure S3.1: Hypothetical inclusion geometry for γ-CD·aescin, in which the cyclodextrin is represented in gray trace with lateral view.

As can be observed from Figure S2.1, this inclusion mode is notoriously similar to the theoretical structure of ‘complex γ1’ calculated by DFT, having the lower formation energy and thus being the most stable geometry predicted for the γ-CD·aescin inclusion complex.
