# Supplementary material for: Association of aescin with β- and γ-cyclodextrins studied by DFT calculations and spectroscopic methods
Source: Beilstein J Nanotechnol. 2017 Feb 3;8:348–57. doi: 10.3762/bjnano.8.37 (PMC5566205; doi:10.3762/bjnano.8.37)
Supplement: File 4 — Studies on the influence of encapsulation on the foam-forming properties of aescin. [file Beilstein_J_Nanotechnol-08-348-s004.pdf]

Supporting Information 4

for

Association of aescin with β- and γ-cyclodextrins studied by DFT calculations and spectroscopic methods

Ana I. Ramos*[1,2], Pedro D. Vaz[3,4], Susana S. Braga[5] and Artur M. S. Silva[5]

Address: [1]CICECO, Complexo de Laboratórios Tecnológicos, Campus Universitário de Santiago, 3810-193 Aveiro, Portugal; [2]Current affiliation: INEGI-FEUP Faculty of Engineering of the University of Porto, Rua Dr. Roberto Frias, 4200-465, Porto, Portugal; [3]CQB, Departamento de Química e Bioquímica, Faculdade de Ciências da Universidade de Lisboa, 1749-016 Lisboa, Portugal; [4]ISIS Neutron & Muon Source, Rutherford Appleton Laboratory, Chilton, Didcot, Oxfordshire OX11 0QX, United Kingdom and [5]QOPNA, Departamento de Química, Universidade de Aveiro, Campus de Santiago, 3810-193 Aveiro, Portugal

Email: Ana I. Ramos - shortinha.sa@gmail.com

* Corresponding author

Studies on the influence of encapsulation on the foam-forming properties of aescin

Experimental details of the foaming assay

Aqueous samples (4 ml) containing either pure aescin (2.5 mg/ml) or mixtures of aescin (also 2.5 mg/ml) and β- or γ-CD in a 1:0.25, 1:0.5, 1:0.75 or 1:1 (mole/mole) were shaken manually for 30 seconds to observe the amount of foam formed in the presence and absence of cyclodextrin. Each experiment was conducted at two different temperature values, ambient temperature (20 °C) and physiological temperature (37 °C).

Results and discussion

As an evidence of the benefits obtained with aescin encapsulation, experiments on foam disruption were performed at room temperature and at 37 °C, shaking solutions of pure aescin or a mixture of aescin:CD, the amount of foam produced and its permanence over time being registered by a series of photographs, shown in the Figure S4.1. It is interesting to note that the introduction of β-CD or γ-CD into the aqueous solutions of aescin (images a-d) did not alter its foam-forming abilities, given that the amount of foam formed is roughly the same as the one occurring in the solution of pure aescin. However, upon 4 h of resting γ-CD seems to contribute to the faster disruption of the foam (Figure e).

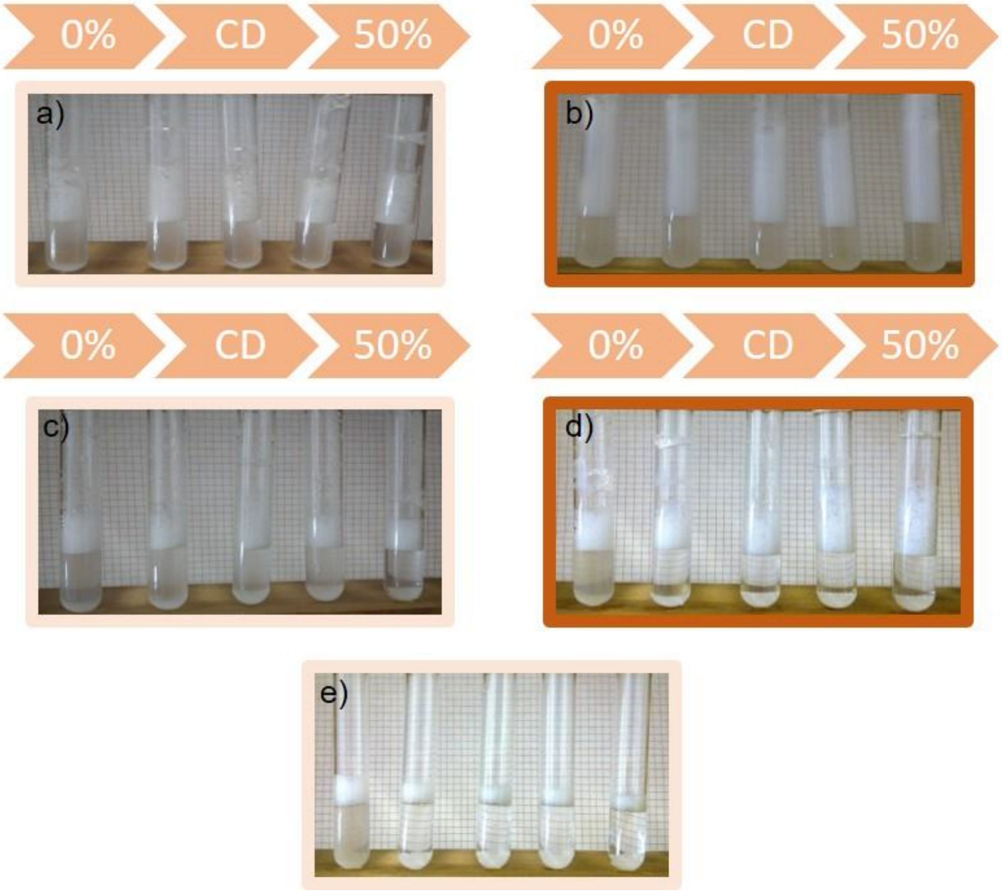

Figure S4.1: Aescin's compared foaming capacity: a) with β-CD at room temperature; b) with β-CD at 37 °C; c) with γ-CD at room temperature; d) with γ-CD at 37 °C; e) with γ-CD at 37 °C after 4 h of foam resting.
